# Shiso ( *Perilla frutescens var. crispa* ) Increases Subjective Pleasantness of Steamed Rice With an Enhanced Sulfur‐Odor

**DOI:** 10.1002/fsn3.72351

**Published:** 2026-09-07

**Authors:** Maaya Toma, Kojiro Ishinaga, Kazuya Saita, Fumiko Kaneko, Hitoshi Okamura

**Affiliations:** ^1^ Graduate School of Biomedical and Health Sciences Hiroshima University Hiroshima Japan; ^2^ Department of Nutrition and Health Promotion Hiroshima Jogakuin University Hiroshima Japan

**Keywords:** olfaction, pleasantness, sensory evaluation, shiso, steamed white rice

## Abstract

When olfactory sensitivity is heightened, sulfur‐related odors can make steamed rice smell unpleasant. This study investigated whether shiso ( *Perilla frutescens var. crispa* ) can enhance the pleasantness of sulfur‐odor–enhanced rice. A sulfur‐odor–enhanced rice model was created by adding raw onion to steamed white rice. Odor intensity was quantified using odor index equivalents, whereas odor quality was assessed using odor similarity patterns. Subjective pleasantness and emotional responses were evaluated in 41 healthy Japanese women using a Visual Analogue Scale (VAS) and the Positive and Negative Affect Schedule (PANAS). The addition of shiso increased perceived pleasantness compared to the unpleasant odor condition (median VAS score: 6.1 vs. 4.1, *p* = 0.029). No changes were observed in odor similarity patterns, whereas odor index equivalents showed a slight decreasing trend; however, these results were interpreted descriptively due to the limited number of measurements. Exposure to the sulfur odor–enhanced rice model reduced positive affect and increased negative affect; however, no significant changes were observed in the shiso‐containing model. The addition of shiso increased perceived pleasantness of steamed rice with enhanced sulfur‐related odors, whereas emotional responses did not change significantly, suggesting that subjective pleasantness and affective responses do not always change in parallel. These findings may inform approaches to improving the pre‐consumption sensory experience of staple foods. Because this pilot study included only healthy Japanese women, the findings cannot be generalized to other populations, including pregnant women and patients undergoing cancer treatment.

## Graphical abstract

Steamed rice can develop a sulfur‐related odor that may reduce its perceived pleasantness. We investigated whether adding shiso to onion‐odor rice could improve sensory pleasantness, using FF‐2020S odor analysis and sensory evaluation with 41 female participants. Shiso addition slightly decreased odor intensity and significantly increased subjective pleasantness (VAS 4.1 to 6.1, *p* = 0.029), with no significant change in mood state.

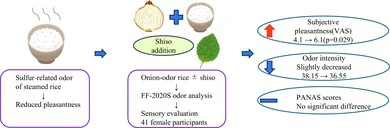

## 1 Introduction

In situations such as cancer treatment or pregnancy, when food preferences change rapidly, not only foods with noticeable odors, such as fish‐based or spiced foods (Coa et al. 2015; Nyaruhucha 2009), but also plain white rice—generally considered to have a mild or neutral odor and taste—can induce aversive reactions and appetite loss (Inagaki 2015; Nyaruhucha 2009; Yoshimoto et al. 2020). However, despite white rice being a staple food in several countries (Muthayya et al. 2014), methods for improving its odor have been underexplored.

A functional magnetic resonance imaging study demonstrated that taste and smell activate common brain regions and interact (De Araujo et al. 2003), indicating that these sensory modalities are closely related. Therefore, whether appetite loss is attributable to taste or smell is difficult to distinguish. Some problems recognized as taste changes may, in fact, originate from alterations in the broader sensory system, including olfaction. In particular, food odor is an important factor that significantly influences food preferences, emotional responses, and subsequent food choice behavior (Proserpio et al. 2017; Zhang and Spence 2023). Furthermore, neurophysiological studies have shown that olfactory information is closely associated with the recognition of flavor characteristics and emotional responses to foods (Kadohisa 2013). For example, odors associated with previous aversive food experiences can elicit disgust and nausea when individuals are subsequently exposed to similar olfactory stimuli (van den Brink et al. 2022). The consumption of unpleasant foods may not only reduce overall preference but also affect odor perception and subsequent food preference, making this an important issue to address.

Multiple volatile compounds are thought to be involved in the unpleasant odor of steamed rice, and sulfur‐containing compounds present in the steam generated during cooking have been suggested as a contributing factor (Yasumatsu 1967). Volatile sulfur compounds are known to cause discomfort even at low concentrations (McGorrin 2011). In contrast, polyphenol‐rich foods have been reported to suppress the odor of sulfur‐containing volatile compounds containing thiol groups (Negishi 1999). Shiso ( *Perilla frutescens var. crispa* ) is an herb rich in polyphenols and characterized by a distinctive aroma. In Japan, it is widely used as a seasoning ingredient, such as in furikake, and pairs well with white rice in everyday meals.

In this study, we investigated whether shiso could improve the perceived unpleasantness of sulfur‐related odors in an onion‐derived sulfur odor–enhanced steamed rice model. Specifically, we examined the effects of adding shiso on odor patterns and intensity, hedonic responses (pleasantness) to the odor, and participants' emotional responses. If the unpleasantness associated with sulfur‐related odors can be alleviated and positive effects on sensory evaluation are observed, this approach may help maintain positive pre‐consumption sensory experiences during periods of changing food preferences. In addition, the findings may provide insights into how improvements in food odor influence hedonic responses and emotional states. This study was conducted as a pilot investigation among healthy adults with appropriate ethical approval to preliminarily assess its effectiveness and validity.

## 2 Materials and Methods

### 2.1 Sampling

Healthy female participants aged 18 years or older were recruited through Hiroshima Jogakuin University (including undergraduate and graduate students) and via public recruitment using the Japan Registry of Clinical Trials (JRCT) from June to September 2024. Previous studies have indicated that olfactory sensitivity and emotional responses can vary depending on sex (McGreevy et al. 2014; Ohla and Lundström 2013). Therefore, this study recruited female participants only, thereby reducing potential variability associated with sex‐related factors. All participants were required to have unobstructed nasal breathing. Prior to the study, a self‐report questionnaire confirmed the absence of food allergies to white rice, onions, or shiso; those with such allergies were excluded. Similarly, participants with olfactory dysfunction following COVID‐19 infection were also excluded.

Sample size was calculated using G*Power 3.1, assuming a paired *t*‐test with an effect size of 0.5 (moderate), 80% power, and a two‐tailed significance level of 5% (Cohen 1988). The calculation indicated that a minimum of 34 participants was required. To account for potential dropouts due to illness or discomfort caused by odor exposure, 41 participants were recruited. As no participants withdrew from the study, all 41 participants completed the study and were included in the final analysis.

This study was approved by the Ethics Review Committee of the Hiroshima Jogakuin University (Certification No. 2023‐6). Written and emailed informed consent was obtained from all participants in compliance with the Declaration of Helsinki. This study was registered with the Japan Registry of Clinical Trials (JRCT) (Registration No. jRCT1060240004). Participants were fully informed about the study procedures and potential risks, were informed that they could withdraw their consent at any time without penalty, and participated voluntarily.

### 2.2 Food Sample Preparation Procedure

Steamed white rice prepared from Saga Prefecture Hinohikari rice (Eguchi et al. 1999) was used. This cultivar has been reported to contain sulfur‐containing compounds; however, whether these compounds contribute to volatile sulfur‐related odor characteristics in cooked rice has not been experimentally verified. A total of 150 g of raw white rice (yielding approximately 330 g of cooked rice) was cooked using a household rice cooker. The perceived odor in cooked white rice is thought to result from multiple volatile sulfur compounds (Zheng et al. 2022); however, particularly in populations experiencing altered food preferences, the specific compounds contributing to the unpleasant perception of rice odor remain unclear. Therefore, in this study, a model odor stimulus was prepared to enhance sulfur‐related odors by adding raw onion, which contains sulfur‐containing volatile compounds, to steamed rice. It should be noted that the aroma profile of onion does not fully correspond to that of cooked rice. Therefore, the present samples were used as an experimental model designed to provide a perceivable sulfur‐related odor stimulus rather than to reproduce the authentic volatile profile of cooked rice. Onion ( *Allium cepa* L.) contains a variety of sulfur‐containing volatile compounds within its essential oil fraction, which contribute to its characteristic aroma (Tang et al. 2026; Kocić‐Tanackov et al. 2012).

Although onion‐derived sulfur compounds differ from those naturally generated in steamed rice, onion was selected as a food‐derived sulfur odor enhancer because it provides a perceivable sulfur‐related odor stimulus suitable for evaluating changes in subjective pleasantness induced by shiso. Two odor conditions were prepared: an onion‐added condition and an onion–shiso added condition, the latter intended to evaluate the effects of shiso on odor reduction. A total of 33 g of raw onion was used to avoid thermal degradation of sulfur‐containing volatile compounds (Jin et al. 2024) and was homogenized using a blender before being mixed uniformly with the steamed rice.

To investigate the potential effects of shiso addition on the perception of onion odor, 1 g of shiso leaves, which are rich in polyphenolic compounds, was finely ground and mixed uniformly with the onion.

The quantities of onion and shiso were determined based on a preliminary pilot study to ensure that the olfactory stimuli were sufficiently perceivable and comparable between conditions. Samples were prepared immediately after cooking the rice, and crushed onion and shiso were added immediately before testing.

Olfactory evaluation was conducted by asking participants to directly inhale the vapor emanating from the rice cooker, and responses were compared between the onion‐added condition and the onion‐shiso added condition.

### 2.3 Measurement Procedures

#### 2.3.1 Objective Analysis of Odor

The odor characteristics of the rice samples were quantified using an odor identification device (FF‐2020S, Shimadzu Corporation, Kyoto, Japan). This device does not conduct a comprehensive chemical analysis of volatile compounds, such as gas chromatography; rather, it is designed to evaluate overall odor patterns by mimicking human olfactory perception. Because the primary objective of this study was to evaluate changes in overall perceived odor characteristics rather than to identify individual volatile compounds, an odor identification device was considered more appropriate than gas chromatography–mass spectrometry (GC–MS) for this purpose and was used to capture changes in the perceived odor profile induced by the addition of shiso, focusing on perceptual differences rather than molecular‐level differences. In addition to the two experimental conditions (rice with onion, and rice with onion and shiso), plain white rice, onion alone, and shiso alone were also measured using the odor identification device as reference samples to aid in the interpretation of the odor patterns. These reference samples were not included in the sensory evaluation and were used only to facilitate comparison with the experimental samples.

#### 2.3.2 Procedure

The test began at around 11:00 AM, approximately 3 h after breakfast. Three rooms were prepared: two sample rooms and a waiting room. Participants were seated randomly in the waiting room, completed a questionnaire, and their baseline affect was assessed using the Positive and Negative Affect Schedule (PANAS).

Participants were first guided to the onion‐only sample room (unpleasant odor condition). Individually, they smelled the steam from a rice cooker while wearing sunglasses and signaled by gesture when they detected the odor. After this assessment, they returned to the waiting room to complete the Visual Analogue Scale (VAS) and PANAS. Following a 5‐min break, they were guided to the onion + shiso sample room (odor reduction condition), where they underwent the same procedure as before, including completion of the questionnaire. The onion‐only condition was always presented first to minimize potential odor cross‐contamination during sample preparation. Thorough ventilation was performed before participants entered the room. The target room temperature was 23°C–26°C, with a relative humidity of 30%–70% (JIS compliant), and the mean room temperature during the experiment was 26.7°C, indicating minimal deviation from the intended range.

Snacking was restricted on the test day, and consumption of sweetened beverages or strongly scented beverages that could affect olfactory function was also prohibited. Only water and tea were permitted. Water was provided throughout the experiment.

### 2.4 Evaluation Scales and Questionnaire

According to Yamamoto (2010), food palatability is defined as a “pleasant emotion”, whereas unpalatability is defined as an “unpleasant emotion”. Emotions are momentary affective states that arise and dissipate over a shorter time span than moods. Therefore, the questionnaire included the VAS to assess pleasantness/unpleasantness and the PANAS to assess affect.

#### 2.4.1 Visual Analogue Scale (VAS)

A 10‐cm Visual Analogue Scale (VAS) was used to assess the subjective pleasantness of each sample, with the left end representing “unpleasant” and the right end representing “pleasant”. The distance from the left end to the mark was measured to one decimal place and converted into a score, with higher scores indicating greater pleasantness.

#### 2.4.2 Positive and Negative Affect Schedule (PANAS)

The PANAS is an emotion assessment scale developed by Watson et al. (Watson et al. 1988) to measure positive (PA) and negative (NA) affect. The Japanese version was translated by Sato and Yasuda (2001). It consists of 16 items, is widely used, and has demonstrated reliability and validity for evaluating emotions in university students. It is rated on a 6‐point scale, with higher scores indicating stronger intensity of the corresponding emotion.

### 2.5 Statistical Analysis

Statistical analyses were performed using IBM SPSS Statistics version 29.0 (IBM Corp., Armonk, NY, USA). The Wilcoxon signed‐rank test was used to compare the two VAS conditions. The Friedman test was used to evaluate changes in PA and NA across the three PANAS conditions (baseline, unpleasant odor, and onion‐shiso condition). Bonferroni correction was used to adjust for multiple comparisons. The significance level was set at *p <* 0.05 (two‐tailed).

## 3 Results

### 3.1 Participant Overview

The age and menstrual status of the 41 participants are summarized in Table 1. Questionnaire data indicated that none of the participants had olfactory dysfunction or allergies to white rice, onions, or shiso used in this study, and thus all were included in the final analyses.

**TABLE 1:** Subject attributes and menstrual status.

Age group	Number of subjects	Number of subjects with menstruation
20s	35	6
30s	4	1
40s	2	1

*Note:* Values represent the number of subjects. Menstrual status indicates subjects reporting regular menstruation at the time of the study.

### 3.2 Similarity Patterns Measured by the Odor Identification Device

Odor similarity, as measured by the odor identification device, represents the similarity of the sample gases relative to a reference gas set at 100%. Similar waveforms can be interpreted as indicating odors of similar quality. The odor identification device's bag could not hold one cup (330 g) of cooked white rice; therefore, 50 g of cooked rice was used to fill the bag. According to Weber–Fechner's law, perceived intensity does not increase proportionally with stimulus intensity (Fechner 1966). Therefore, the amounts of onion (33 g) and shiso (1 g) were kept consistent with the subjective evaluation procedure, regardless of the rice weight.

The odor identification device was used to visualize odor‐pattern similarities. Quantitative similarity indices suitable for statistical comparison were not obtained in the present study; therefore, the radar plots were interpreted qualitatively.

The odor similarity measurements for the samples used in the sensory evaluation (white rice + onion and white rice + onion + shiso) were performed three times for each sample, and the mean values were used for analysis. As reference samples, white rice alone, onion alone, and shiso alone were also measured once to assist in the interpretation of the odor patterns of the mixed samples. The amount of shiso in the mixed samples (1 g) was defined according to the sensory evaluation protocol, whereas shiso alone (5 g) was measured solely as an auxiliary reference to aid the qualitative interpretation of odor‐pattern characteristics. For plain white rice, a stable response pattern was not obtained under the present conditions due to measurement variability; therefore, the results were interpreted qualitatively and used only as a reference for odor‐pattern interpretation.

**FIGURE 1:**
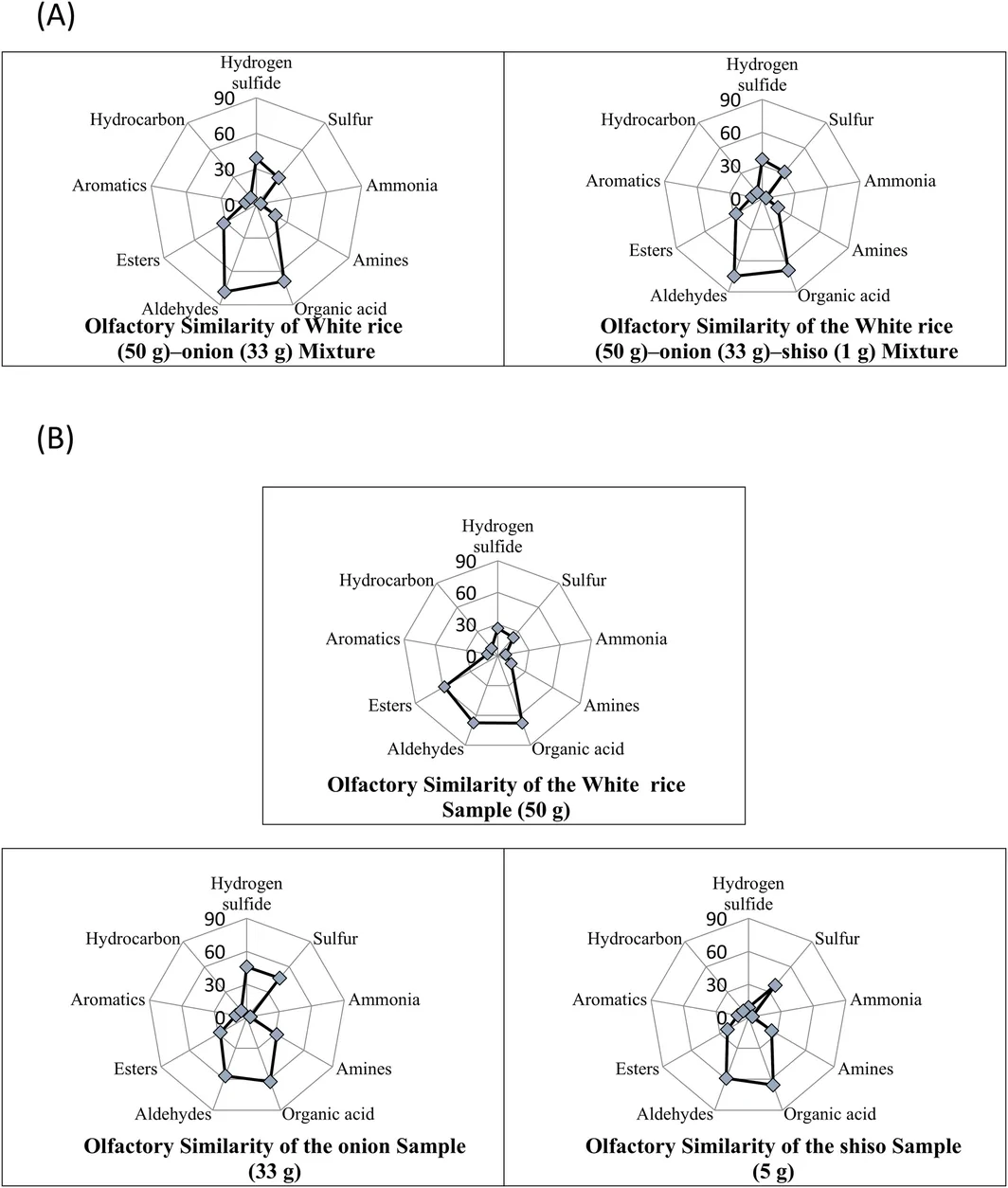
Odor similarity patterns measured using the FF‐2020S odor identification device. (A) Odor similarity patterns of the mixed samples used in the subjective evaluation (white rice + onion and white rice + onion + shiso), expressed as the mean of three measurements. (B) Representative odor similarity patterns of the individual ingredients (plain white rice, onion, and shiso), each obtained from a single measurement and presented as reference data. The radar plots illustrate the similarity of each sample to predefined odor categories generated by the FF‐2020S odor identification device. Plain white rice showed unstable measurements under the present conditions; therefore, its odor similarity pattern is presented only as a representative reference.

The odor pattern of onion alone exhibited an hourglass shape with large upper and lower peaks and an overall bulging profile. In contrast, the odor pattern of white rice + onion was also hourglass‐shaped but elongated, with comparatively smaller upper and lower peaks, indicating that the mixed sample exhibited a different odor pattern from that of the individual ingredient samples. Likewise, the odor pattern of white rice + onion + shiso differed from that of shiso alone, suggesting that the odor patterns of the mixed samples were distinct from those of the individual ingredients. Meanwhile, the odor patterns of white rice + onion and white rice + onion + shiso were both elongated and hourglass‐shaped, and no obvious qualitative differences were observed between them (Figure 1A).

The contribution of plain white rice to sulfur‐related odor intensity was relatively low; however, even at low concentrations (McGorrin 2011), sulfur‐related odors may still be perceived as unpleasant and may contribute to the overall perceptual odor profile (Figure 1B).

### 3.3 Measurement of Odor Index Equivalents

Odor index equivalents are values calculated based on a threshold correction simulating human olfaction, indicating the odor intensity of a sample. The samples used for the sensory evaluation (white rice + onion and white rice + onion + shiso) were measured three times each, and the mean values were used for analysis. The results are shown in Table 2. Due to the limited number of measurements and variability in odor measurements across measurement days, inferential statistical analysis was not performed. A slight decrease in odor index equivalents was observed after the addition of shiso.

**TABLE 2:** Odor index equivalent values under two conditions.

Type of food (in triplicate)	Odor index equivalent value (mean ± SD)
White rice 50 g + onion 33 g	38.15 ± 4.90
White rice 50 g + onion 33 g + shiso 1 g	36.55 ± 5.82

*Note:* Odor index equivalent values were determined by odor measurement under the present analytical conditions.

### 3.4 Subjective Evaluation of Pleasantness/Unpleasantness via the VAS

During the olfactory test, the white rice in the cooker was maintained at approximately 60°C. The median VAS scores for the unpleasant odor sample (white rice + onion) and the shiso‐containing sample (white rice + onion + shiso) were 4.1 and 6.1, respectively, indicating a significant improvement in pleasantness with shiso addition (*p* = 0.029; white rice + onion: 4.1 [3.3–7.1], white rice + onion + shiso: 6.1 [4.5–7.2], *z* = 2.178, *r* = 0.34) (Table 3).

**TABLE 3:** Subjective evaluation (VAS) of pleasantness/unpleasantness of odor with and without shiso addition.

Sample	Pleasantness/Unpleasantness, VAS, median (quartile range)	*p*
White rice 50 g + onion 33 g	4.1 (3.3–7.1)	0.029
White rice 50 g + onion 33 g + shiso 1 g	6.1 (4.5–7.2)	

*Note:* Values are presented as median (interquartile range). *p*‐values were calculated using the Wilcoxon signed‐rank test.

Abbreviation: VAS, visual analog scale.

### 3.5 Emotional Changes Using the PANAS

The medians and interquartile ranges of the PANAS scores are shown in Table 4.

**TABLE 4:** Median PANAS scores at 3 time points (interquartile range).

	Baseline	50 g White rice +33 g onion	50 g White rice +33 g onion +1 g shiso	*p*	Post hoc (Bonferroni)
Positive affect	19.00 (14.50–23.50)	15.00 (11.00–22.00)	14.00 (10.50–19.00)	< 0.001**	Baseline vs. onion (*p* = 0.004**)
Active	4.00 (3.00–4.00)	2.00 (1.00–4.00)	3.00 (1.00–4.00)	< 0.001**	Baseline vs. onion (*p* < 0.001**)
Proud	2.00 (1.00–3.00)	1.00 (1.00–2.00)	1.00 (1.00–2.00)	0.169	
Strong	2.00 (1.00–2.50)	1.00 (1.00–2.00)	1.00 (1.00–2.00)	0.650	
Determined	3.00 (2.00–4.00)	2.00 (1.00–3.00)	2.00 (1.00–3.00)	< 0.001**	Baseline vs. onion (*p* = 0.003**)
Attentive	1.00 (1.00–2.00)	1.00 (1.00–2.00)	1.00 (1.00–2.00)	0.289	
Excited	4.00 (3.00–4.50)	3.00 (2.00–4.00)	3.00 (1.00–4.00)	0.004**	
Alert	1.00 (1.00–3.00)	1.00 (1.00–2.00)	1.00 (1.00–2.00)	0.159	
Inspired	1.00 (1.00–2.50)	1.00 (1.00–2.00)	1.00 (1.00–2.00)	0.089	
Negative affect	11.00 (8.00–13.00)	11.00 (8.50–17.50)	11.00 (8.00–17.00)	0.477	
Nervous	1.00 (1.00–1.00)	1.00 (1.00–2.00)	1.00 (1.00–2.00)	0.002**	Baseline vs. onion (*p* = 0.001**)
Afraid	1.00 (1.00–1.00)	1.00 (1.00–2.00)	1.00 (1.00–2.00)	< 0.001**	Baseline vs. onion (*p* < 0.001**)
Scared	1.00 (1.00–1.50)	1.00 (1.00–2.00)	1.00 (1.00–2.00)	0.080	
Upset	2.00 (1.00–3.00)	2.00 (1.00–3.00)	2.00 (1.00–3.00)	0.508	
Jittery	1.00 (1.00–2.00)	1.00 (1.00–2.00)	1.00 (1.00–2.00)	0.926	
Hostile	1.00 (1.00–2.00)	1.00 (1.00–2.00)	1.00 (1.00–2.00)	0.988	
Ashamed	1.00 (1.00–1.50)	1.00 (1.00–1.50)	1.00 (1.00–1.00)	0.217	
Irritable	1.00 (1.00–1.00)	1.00 (1.00–1.00)	1.00 (1.00–1.00)	0.141	

*Note:* Values are presented as median (interquartile range). *p*‐values were calculated using the Friedman test. Post hoc pairwise comparisons were performed using the Bonferroni correction when the Friedman test was significant. A significance level of *p* < 0.0167 was used for the Bonferroni‐adjusted post hoc comparisons. Only statistically significant pairwise comparisons are shown.

Abbreviation: PANAS, Positive and Negative Affect Schedule.

** *p* < 0.01.

Friedman tests revealed significant differences in the PA total score and the items “active”, “determined”, and “excited”, as well as the NA items “nervous” and “afraid”. Post hoc Bonferroni multiple comparisons showed that the PA total score significantly decreased from baseline to the white rice + onion condition (*p = 0*.004; Baseline: 19 [14.5–23.5], white rice + onion: 15 [11–22], white rice + onion + shiso: 14 [10.5–19.0], *z* = −2.882, *r* = −0.45). Similarly, the score for “active” significantly decreased from baseline to the white rice + onion condition (*p < 0*.001; Baseline: 4 [3–4], white rice + onion: 2 [1–4], white rice + onion + shiso: 3 [1–4], *z* = −3.501, *r* = −0.55), and the score for “determined” significantly decreased (*p* = 0.003; Baseline: 3 [2–4], white rice + onion: 2 [1–3], white rice + onion + shiso: 2 [1–3], *z* = −3.007, *r* = −0.47). For the NA items, the score for “nervous” significantly increased from baseline to the white rice + onion condition (*p* = 0.001; Baseline: 1 [1–1], white rice + onion: 1 [1–2], white rice + onion + shiso: 1 [1–2], *z* = −3.213, *r* = −0.50), and the score for “afraid” also significantly increased (*p* < 0.001; Baseline: 1 [1–1], white rice + onion: 1 [1–2], white rice + onion + shiso: 1 [1–2], *z* = −3.363, *r* = −0.53). No significant differences were observed between the white rice + onion and white rice + onion + shiso conditions in any emotional variables (all *p* > 0.05).

These findings indicate that exposure to the unpleasant odor decreased positive affect and increased negative affect, whereas the addition of shiso did not produce statistically detectable improvements in emotional responses.

## 4 Discussion

### 4.1 Changes in Subjective Pleasantness of Sulfur‐Rrelated Off‐Odors Following Shiso Addition

This study investigated the effects of adding shiso on perceived pleasantness and emotional responses to an onion‐derived sulfur odor–enhanced steamed rice model.

Regarding the effect of shiso on odor perception in the sulfur odor–enhanced rice model, the VAS results (Table 3) indicated greater hedonic ratings, suggesting that shiso enhanced the perceived pleasantness of sulfur odor–enhanced steamed rice.

A key finding of this study is that odor intensity (odor index–equivalent values) showed a slight decreasing trend, whereas subjective unpleasantness showed a significant improvement. This discrepancy suggests that, whereas objective measurements primarily reflect odor intensity, subjective pleasantness is influenced not only by odor strength but also by cognitive and physiological factors, such as expectation based on contextual information, sleep status, circadian rhythm, and individual mood states (Piqueras‐Fiszman and Spence 2015; Shanahan and Kahnt 2022). Therefore, even when changes in measured odor intensity are small, perceived pleasantness may still change.

However, this study focused on sensory evaluation of odor pleasantness and did not include chemical analysis of volatile compounds (e.g., GC–MS). Thus, it remains unclear whether shiso directly altered sulfur‐containing volatile compounds, or whether it affected olfactory perception and/or other odor components. Future studies using chemical analytical approaches are required to clarify the underlying mechanisms.

In addition, because a randomized crossover design was not employed, potential order effects, such as sensory adaptation, habituation, and expectancy bias, cannot be fully excluded. Previous studies have reported that polyphenol‐rich food components may interact with sulfur‐containing compounds and modify odor perception. Therefore, the effects of polyphenol‐rich ingredients such as shiso on the hedonic perception of sulfur‐related odors in foods warrant further investigation. However, both the efficacy and the underlying mechanisms of such effects should be examined under real consumption conditions and in combination with chemical analyses.

### 4.2 Emotional Modulation by Shiso Odor

Olfactory stimuli differ from other sensory modalities in that they are closely linked to emotional processing (Turner et al. 1978, 1980). Shiso has been used in the form of essential oils, and olfactory stimulation has been reported to induce physiological relaxation by significantly reducing oxyhemoglobin concentration in the prefrontal cortex (Igarashi et al. 2014). Moreover, shiso has a history of use in traditional herbal medicine for the treatment of psychological stress; its main aroma compounds—perillaldehyde and limonene—have been reported to exert central nervous system inhibitory effects, prolong sleep, and induce relaxation (do Vale et al. 2002; Qiu et al. 2021).

However, in this study, exposure to the onion‐derived sulfur odor–enhanced rice model was associated with increases in some negative affective states and decreases in positive affect, suggesting that unpleasant olfactory stimulation may have transient effects on emotional states. Given that olfaction is closely linked to emotion, exposure to unpleasant odors may induce temporary increases in negative affect and decreases in positive affect. Nevertheless, although the addition of shiso to sulfur odor–enhanced rice improved perceived pleasantness, no significant changes in emotional responses measured using the PANAS were observed. These results are consistent with previous studies examining short‐term emotional responses to odors, which have suggested that perceptual evaluations of odors and emotional states measured by PANAS do not necessarily show a consistent correspondence (Kuesten et al. 2014). A similar trend may have been observed in the present study. Since the affective value of olfactory stimuli is known to be shaped by experience and memory (Kadohisa 2013), it is possible that shiso improved perceived odor pleasantness without producing changes large enough to be detected at the level of general emotional states measured by the PANAS. In addition, the relatively mild nature of food‐related odor stimuli may have contributed to these findings. Furthermore, the short duration of odor exposure and the potential burden associated with repeated completion of the 16‐item PANAS questionnaire may also have influenced the results. Overall, these findings suggest that improvements in perceived odor pleasantness may not necessarily translate into measurable changes in broader emotional states as assessed by the PANAS. Future studies should examine these relationships under stronger stimulus conditions and/or longer exposure durations. The incorporation of physiological measures, optimization of stimulus conditions, and the use of multimodal assessment approaches are warranted.

### 4.3 Implications for Food Design and Culinary Applications

The findings of this study provide preliminary evidence regarding improvements in perceived odor pleasantness using food ingredients, suggesting potential applications for future food design and cooking methods. Odor is generally evaluated along a hedonic dimension of pleasantness and unpleasantness (Zarzo 2008; Schiffman 1974), and food odors are closely associated with food preference and appetite (Zhang and Spence 2023). In the present study, an approximately 2‐point improvement in VAS scores was observed, indicating that participants perceived the odor as more pleasant. These results suggest that shiso addition may have practical relevance from the perspectives of consumer acceptability and dietary support. However, since the present study was conducted under laboratory conditions, its implications for perceived acceptability in real‐world eating environments should be interpreted with caution.

However, this study was conducted with healthy young female participants; therefore, the results cannot be directly generalized to clinical populations or specific patient groups, such as pregnant women or individuals with cancer. Nevertheless, this study may be considered an exploratory investigation into food ingredients that influence olfactory pleasantness, and further research is required to examine different populations and individuals with diverse food preference characteristics.

Although white rice was used as the test food in this study, previous reports suggest that individuals with heightened olfactory sensitivity may also experience unpleasantness toward meat, seafood, and high‐fat foods. Future research is therefore needed to clarify the common characteristics of unpleasant food odors. For practical application, further evaluation under diverse conditions, assessment of combinations with a wider range of foods, and sensory testing under real eating environments will also be necessary.

### 4.4 Study Limitations

The present study has several limitations.

First, chemical characterization of sulfur‐related odor compounds in the cooked rice was not performed. Therefore, it remains unclear whether the observed effects were due to changes in sulfur compounds themselves, other volatile compounds, or changes in subjective odor perception. Although this study focused on sensory evaluation of odor changes and suggested improvements in perceived pleasantness, it did not directly investigate underlying mechanisms. Future studies incorporating quantitative chemical analyses, such as GC–MS, are required to address this issue.

Second, the order of stimulus presentation was fixed. A randomized crossover design could not be implemented because of concerns regarding odor cross‐contamination during sample preparation using a mixer. As a result, potential order effects, such as sensory adaptation, habituation, fatigue, and expectancy bias, cannot be fully excluded. Future studies should establish sample preparation methods that prevent cross‐contamination and employ randomized experimental designs to confirm these findings.

Third, only a single level of shiso addition was examined. Although the dosage was determined based on a pilot study and typical usage levels, dose–response relationships and optimal concentrations were not investigated. Future studies should examine multiple concentrations to determine threshold effects and optimal ranges.

Fourth, there were inconsistencies in sample conditions between instrumental odor measurements and sensory evaluations. Due to methodological constraints, the amount of white rice differed between instrumental analysis and sensory testing. Therefore, differences in sample weight and measurement conditions may have influenced volatile release and odor intensity, limiting direct comparison between instrumental and sensory results. Future studies should standardize sample conditions across measurement methods as much as possible.

In addition, the onion‐based sulfur odor model used in this study does not fully replicate the chemical composition of off‐odors in cooked rice. Therefore, the findings should be interpreted as sensory responses obtained under an experimental sulfur‐odor–enhanced model.

Moreover, although an improvement in perceived pleasantness following shiso addition was observed, the present study could not determine whether this effect was attributable to changes in volatile compounds, sensory adaptation, expectancy effects, or other factors influencing subjective odor evaluation. In addition, participants' familiarity with and preference for white rice, onion, and shiso were not assessed. Because these factors may influence odor perception and hedonic evaluation, their potential contribution to the observed responses cannot be excluded. Future studies should incorporate assessments of participants' familiarity and preferences, as well as additional control conditions, such as a plain rice condition, an onion‐only condition, and a shiso‐only condition, to provide a more comprehensive interpretation of the sensory findings. Furthermore, the study population was limited to healthy Japanese women, and the generalizability of the findings across sexes and cultural backgrounds has not been established. Therefore, caution is required when extrapolating these findings to other populations, particularly clinical groups with olfactory hypersensitivity, such as patients with cancer or pregnant women.

Future research should incorporate physiological measures, expand the study population, and examine responses under conditions that more closely reflect real‐world eating environments.

## 5 Conclusions

This pilot study focused on unpleasant odors in cooked rice that may reduce perceived sensory pleasantness prior to eating, particularly in individuals with heightened olfactory sensitivity, and examined the effects of adding shiso on perceived odor pleasantness. Sensory evaluation using the VAS and emotional assessment using the PANAS were conducted among healthy Japanese female participants. The results showed that the addition of shiso improved perceived pleasantness as measured by the VAS, whereas no significant changes were observed in emotional responses. These findings suggest that shiso may enhance olfactory pleasantness at the pre‐consumption stage.

However, this study was a pilot investigation using a fixed‐order design and a sulfur‐odor–enhanced model, and the participants were limited to healthy Japanese women. Therefore, caution is required when generalizing the results, and direct application of the findings to clinical populations with olfactory hypersensitivity is not appropriate.

Before practical application, future studies should incorporate randomized designs, quantitative chemical analyses (e.g., GC–MS) to clarify the underlying mechanisms, and more diverse participant groups to further examine the reproducibility and generalizability of these findings.

## Author Contributions

**Maaya Toma:** conceptualization, methodology, validation, formal analysis, investigation, data curation, writing – original draft, writing – review and editing, visualization. **Kojiro Ishinaga:** conceptualization, methodology, validation, resources, project administration. **Kazuya Saita:** conceptualization, methodology, validation, writing – original draft, writing – review and editing, project administration, visualization. **Fumiko Kaneko:** conceptualization, methodology, validation, writing – original draft, writing – review and editing, visualization, project administration. **Hitoshi Okamura:** conceptualization, methodology, validation, project administration, supervision, writing – original draft, writing – review and editing.

## Funding

This work was supported by operational funding from Hiroshima University.

## Disclosure

Author approval and responsibility statement: All authors have read and approved the final version of the manuscript. The corresponding author had full access to all of the data in this study and takes complete responsibility for the integrity of the data and the accuracy of the data analysis.

## Ethics Statement

This study was approved by the Ethics Review Committee of the Hiroshima Jogakuin University (Certification No. 2023‐6). The study was conducted in compliance with the Declaration of Helsinki. This study was registered with the Japan Registry of Clinical Trials (JRCT) (Registration No. jRCT1060240004).

## Consent

Written informed consent was obtained from all study participants.

## Conflicts of Interest

The authors declare no conflicts of interest.

## Data Availability

The data that support the findings of this study are available from the corresponding author upon reasonable request.
